# Supplementary material for: High throughput screening identifies SOX2 as a super pioneer factor that inhibits DNA methylation maintenance at its binding sites
Source: Nat Commun. 2021 Jun 7;12:3337. doi: 10.1038/s41467-021-23630-x (PMC8184831; doi:10.1038/s41467-021-23630-x)
Supplement: Supplementary file 1 — Supplementary Info [file 41467_2021_23630_MOESM1_ESM.pdf]

Supplementary information

High throughput screening identifies SOX2 as a Super Pioneer Factor that inhibits DNA methylation maintenance at its binding sites.

Ludovica Vanzan, Hadrien Soldati, Victor Ythier, Santosh Anand, Simon M.G. Braun, Nicole Francis, Rabih Murr

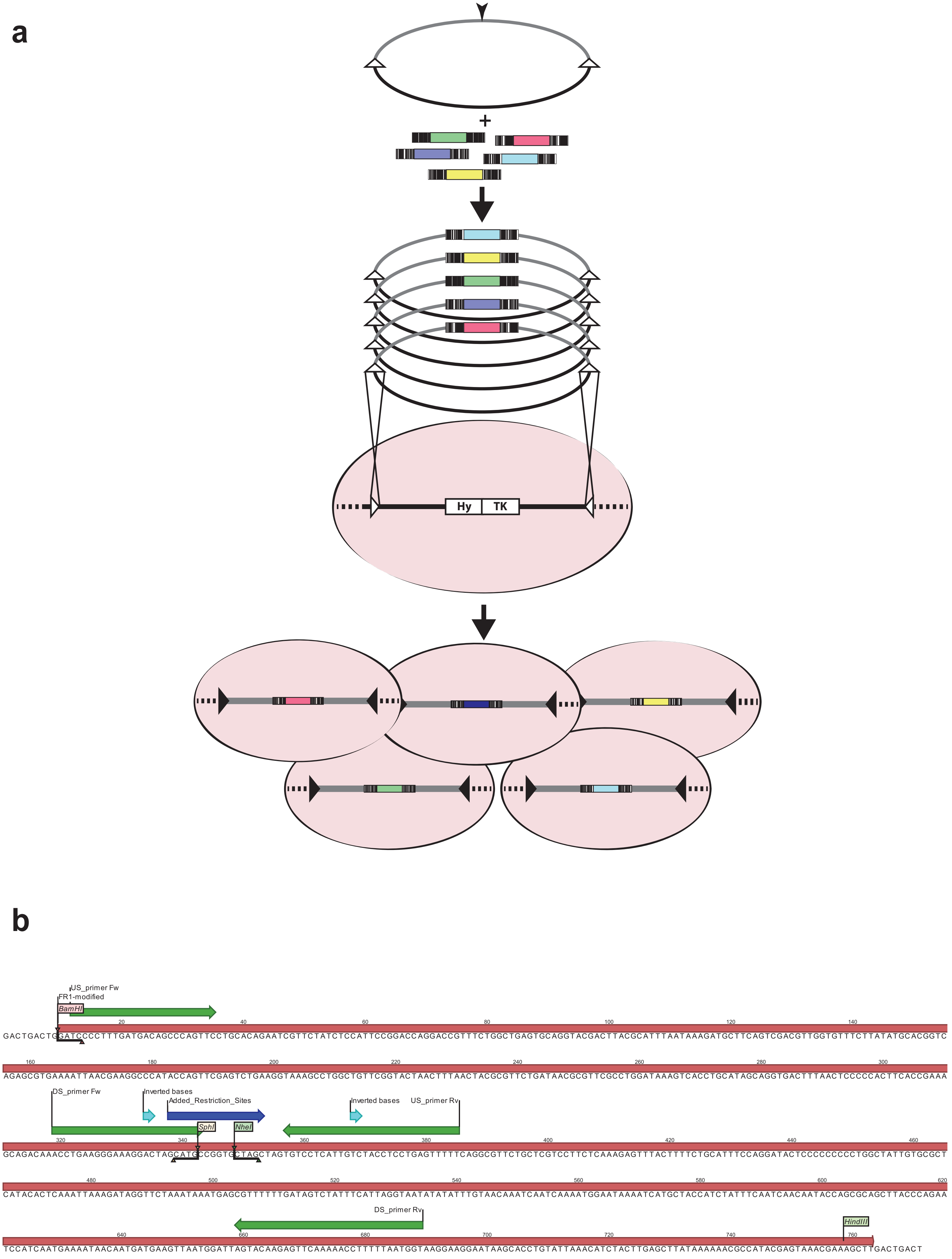

Supplementary Figure 1

Supplementary Figure 1. Experimental approach.

(**a**) Schematic representation of the Hi-TransMet approach. A library of targeting plasmids each containing the same bacterial DNA fragment, (FR1) represented by a grey line, but a different motif (colored rectangles) and barcodes (dashed boxes) and flanked by *LoxP* sites (triangles) were transfected into ESCs containing the RMCE site together with a plasmid expressing CRE recombinase. This leads to the replacement of the selection cassette (Hygromycin/Thymidine Kinase) by the bacterial fragment. Ganciclovir treatment selects the cells that underwent recombination. Genomic DNA is extracted from successfully recombined cells and treated with sodium bisulfite. (**b**) Sequence of the bacterial fragment FR1 used in this study. Primer sequences for Bisulfite PCR and library preparation are indicated in green. US_Primer is the upstream primer pair, DS_Primer is the downstream primer pair (please refer to text for further details). Edits to the original sequence[42] are indicated in dark blue (additions) and light blue (changes of position). The fragment was inserted into the RMCE donor plasmid by directional cloning using the restriction enzymes BamHI and HindIII (flags); motifs were later inserted by directional cloning with SphI and NheI (flags).

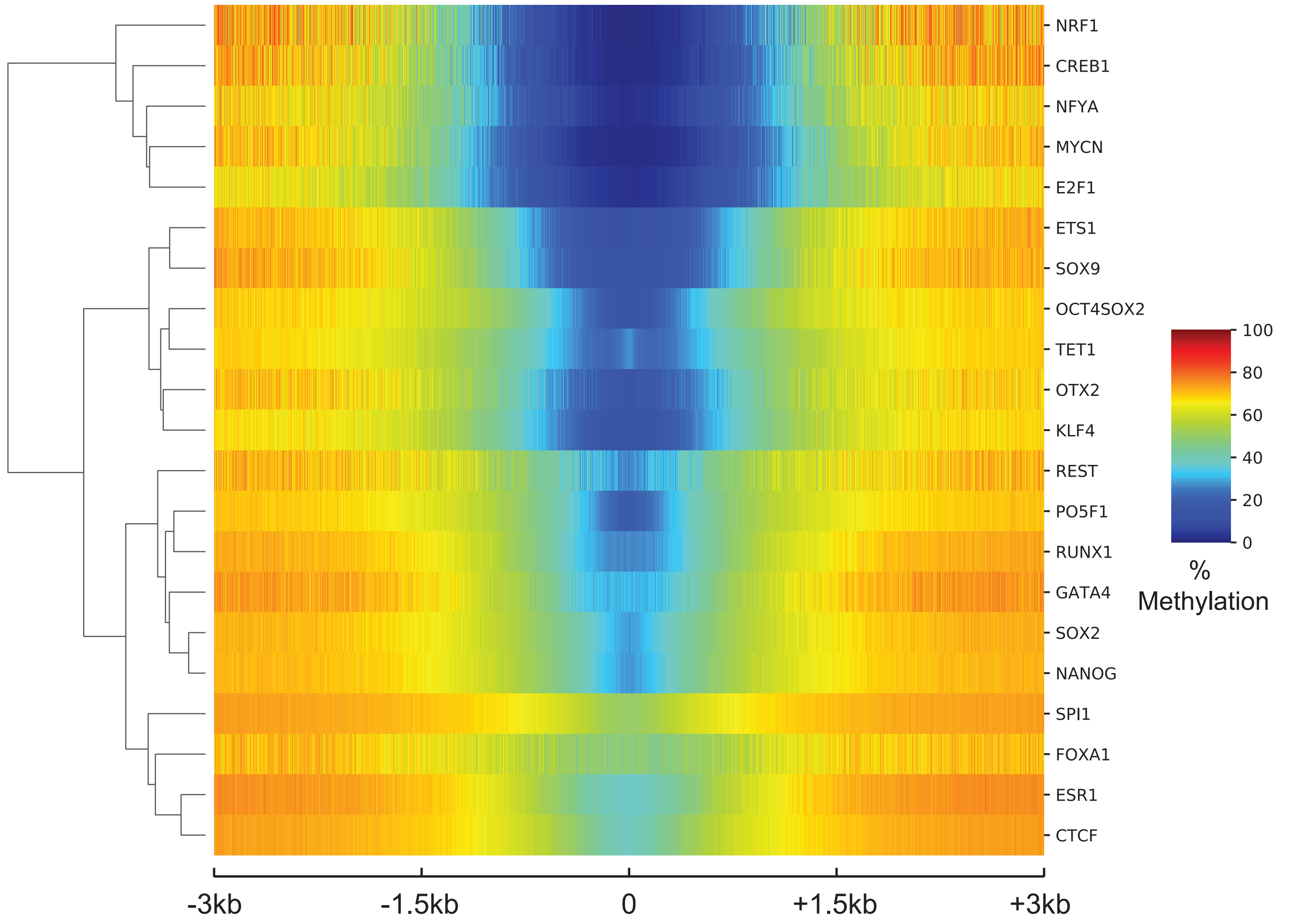

Supplementary Figure 2

Supplementary Figure 2. Common endogenous PF-binding sites exhibit low methylation levels in ESCs.

Heat map of methylation levels 3kb around the center of mouse aggregate cistromes, the peaks of routinely identified binding sites for different PFs in several cell types.

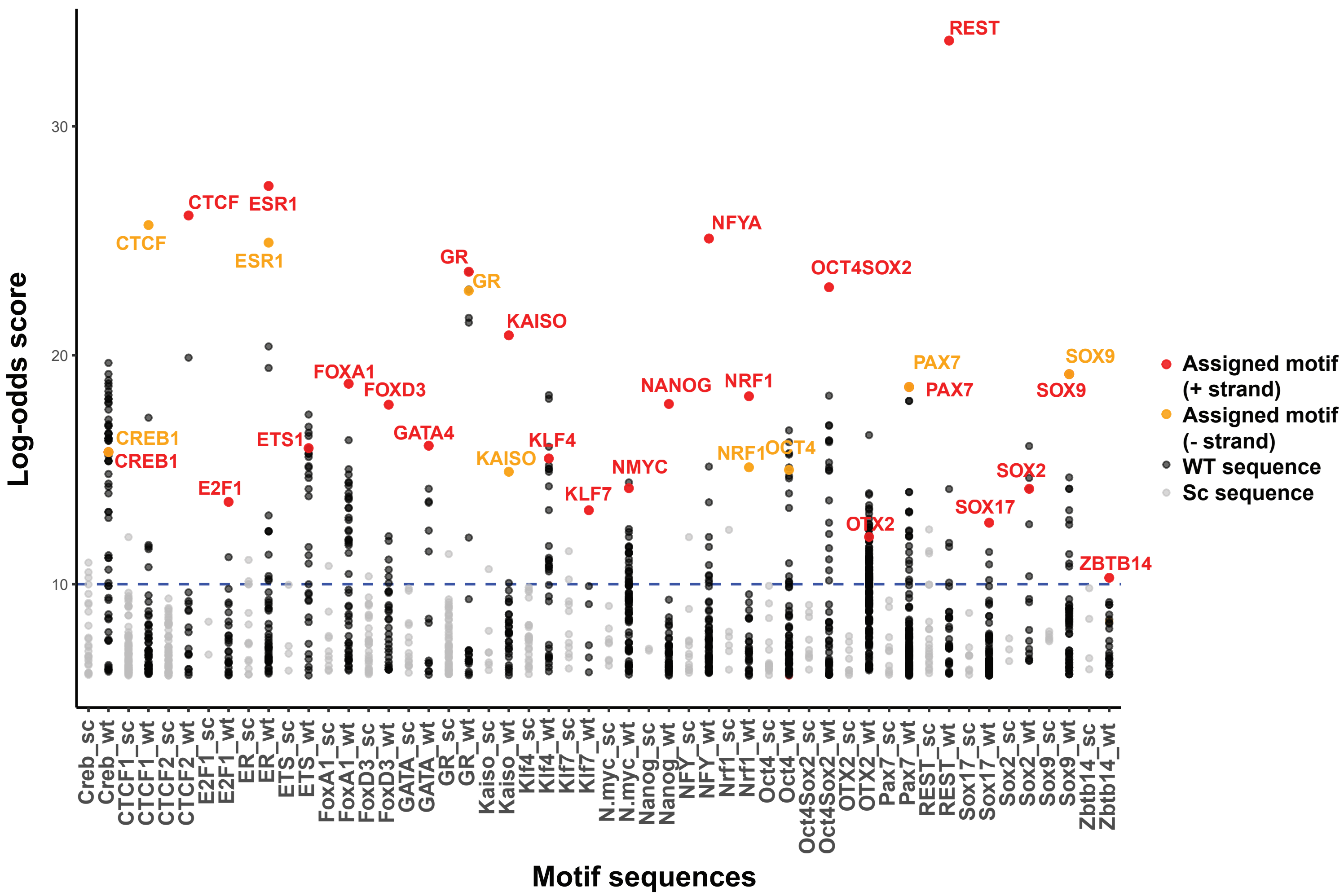

Supplementary Figure 3

Supplementary Figure 3. ***In silico*** **analysis of different restriction site-barcode-motif combinations.**

Score of transcription factors predicted using HOMER tool for all restriction site-barcode-motif combinations. Black and gray dots are all TFBS predicted for WT and Sc sequences, respectively. TFBSs assigned to WT sequences are represented by red (top strand) and orange (bottom strand) dots.

a

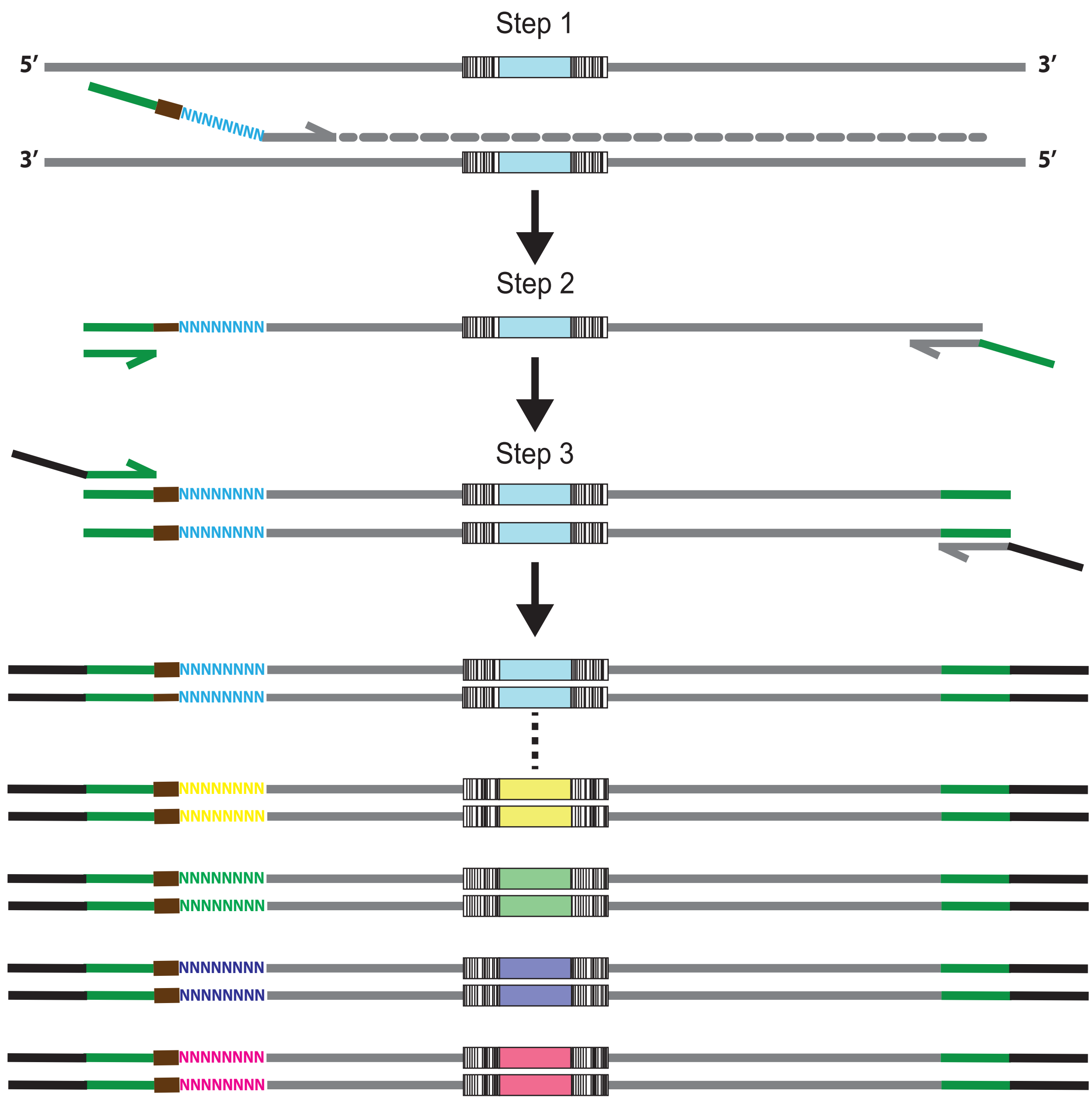

b

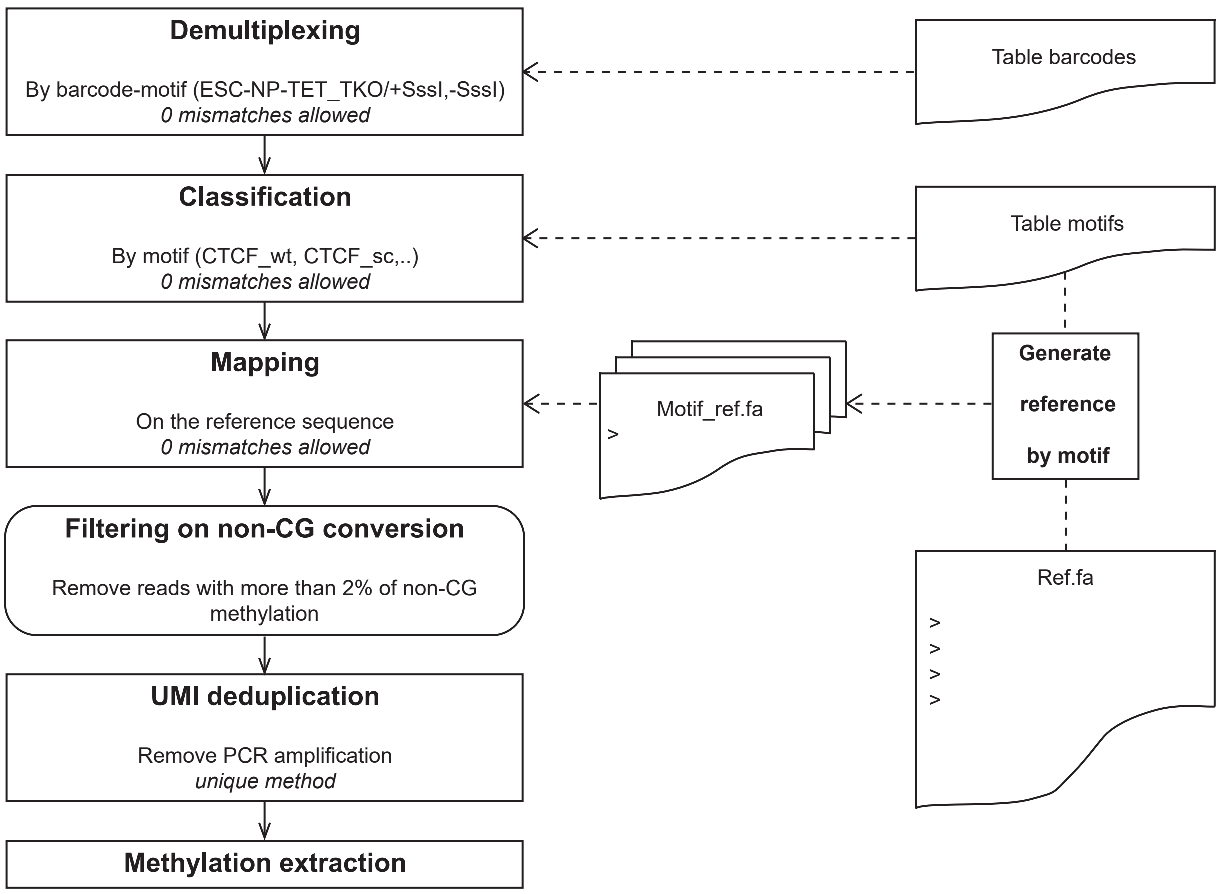

Supplementary Figure 4

Supplementary Figure 4. Hi-TransMet library preparation and molecular barcoding.

(**a**) Hi-TransMet library preparation. Step 1, UMI assignment. A target-specific reverse primer, including a UMI tag (colored Ns) and a library barcode (black) is annealed to the bisulfite-converted DNA and the target sequence is extended. Step 2, non-barcoded amplification. A short PCR amplification is performed using forward target-specific primers and a reverse universal primer. Step 3, addition of sequencing adapters by PCR amplification. Unused primers and primer dimers are removed between each step. The region of interest surrounding the motifs is PCR amplified using two sets of universal primers, upstream and downstream of the motifs, covering about 500 bp flanking the binding sites. (**b**) Overview of the bioinformatic pipeline.

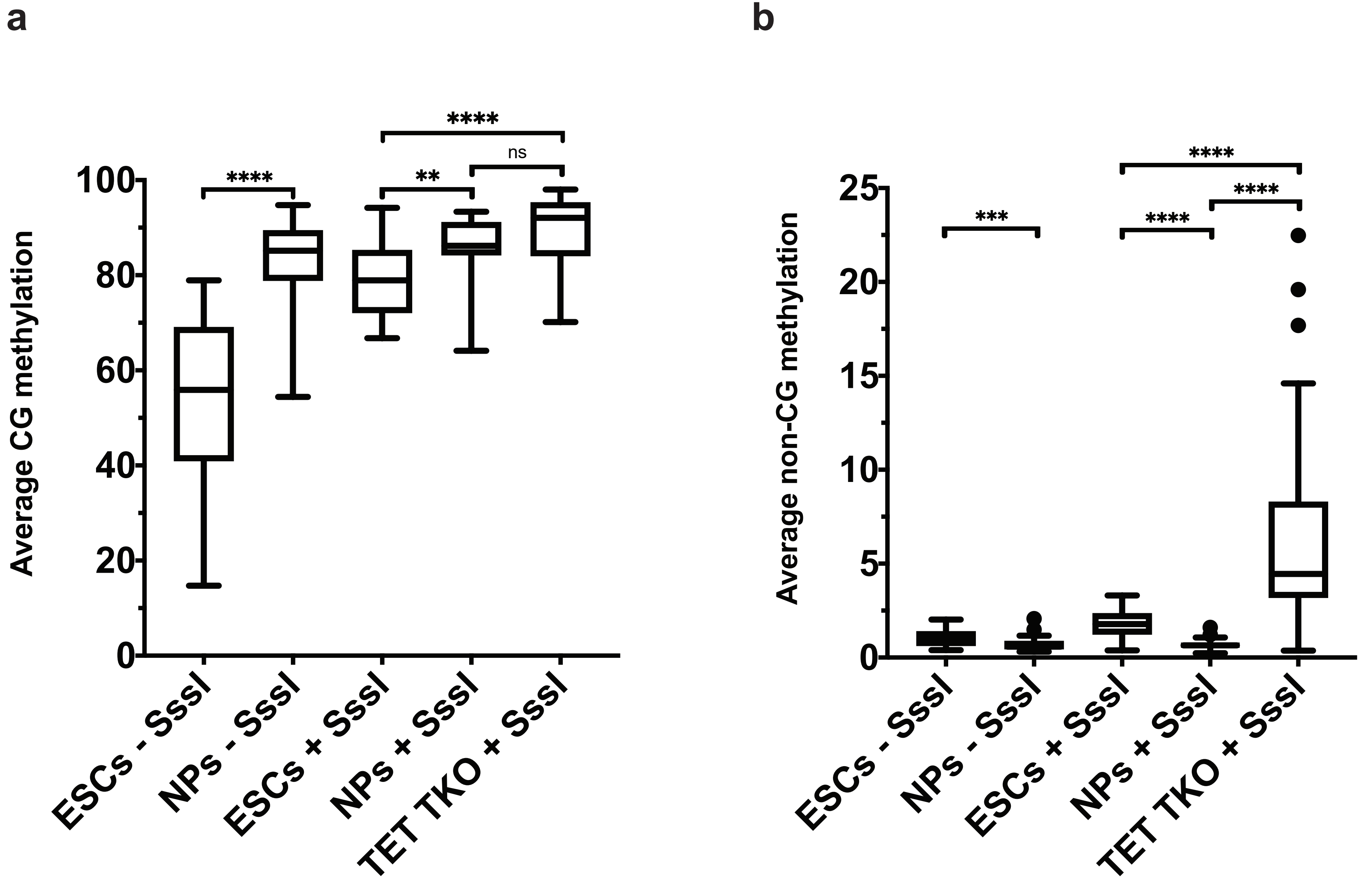

Supplementary Figure 5

Supplementary Figure 5. Overall methylation levels on FR1 fragment in WT ESCs, NPs and TET TKO ESCs.

(**a**) Mean ratio of CpG methylation in WT ESCs, NPs and TET TKO ESCs measured at FR1 fragments containing Sc motifs (n=26). P-values (two-tailed paired t-test, **p<0.01, ****p<0.0001): NPs/-SssI vs ESCs/-SssI: p=1.59E-07; NPs/+SssI vs ESCs/+SssI: p=0.0025; TET-TKO/+SssI vs ESCs/+SssI: p=1.01E-0.5; TET-TKO/+SssI vs NPs/+SssI: p=0.1527. (**b**) Mean ratio of non CpG methylation (CHG+CHH) in WT ESCs, NPs and TET TKO ESCs measured at FR1 fragments containing Sc motifs (n=26). P-values (two-tailed paired t-test, ***p<0.001, ****p<0.0001): NPs/-SssI vs ESCs/-SssI: p=0.0010; NPs/+SssI vs ESCs/+SssI: p=1E-07; TET-TKO/+SssI vs ESCs/+SssI: p=0.0002; TET-TKO/+SssI vs NPs/+SssI: p=2.65E-05. In both panels (**a**) and (**b**) data are represented as box plots where the middle line is the median, the lower and upper hinges correspond to the 25th and the 75th percentiles, the lower whisker extends from the lower hinge to the smallest value and the upper whisker extends from the upper hinge to the biggest value not bigger than the 75th percentile plus 1.5 IQR (inter-quartile range). For all panels, source data are provided as a Source data file.

a

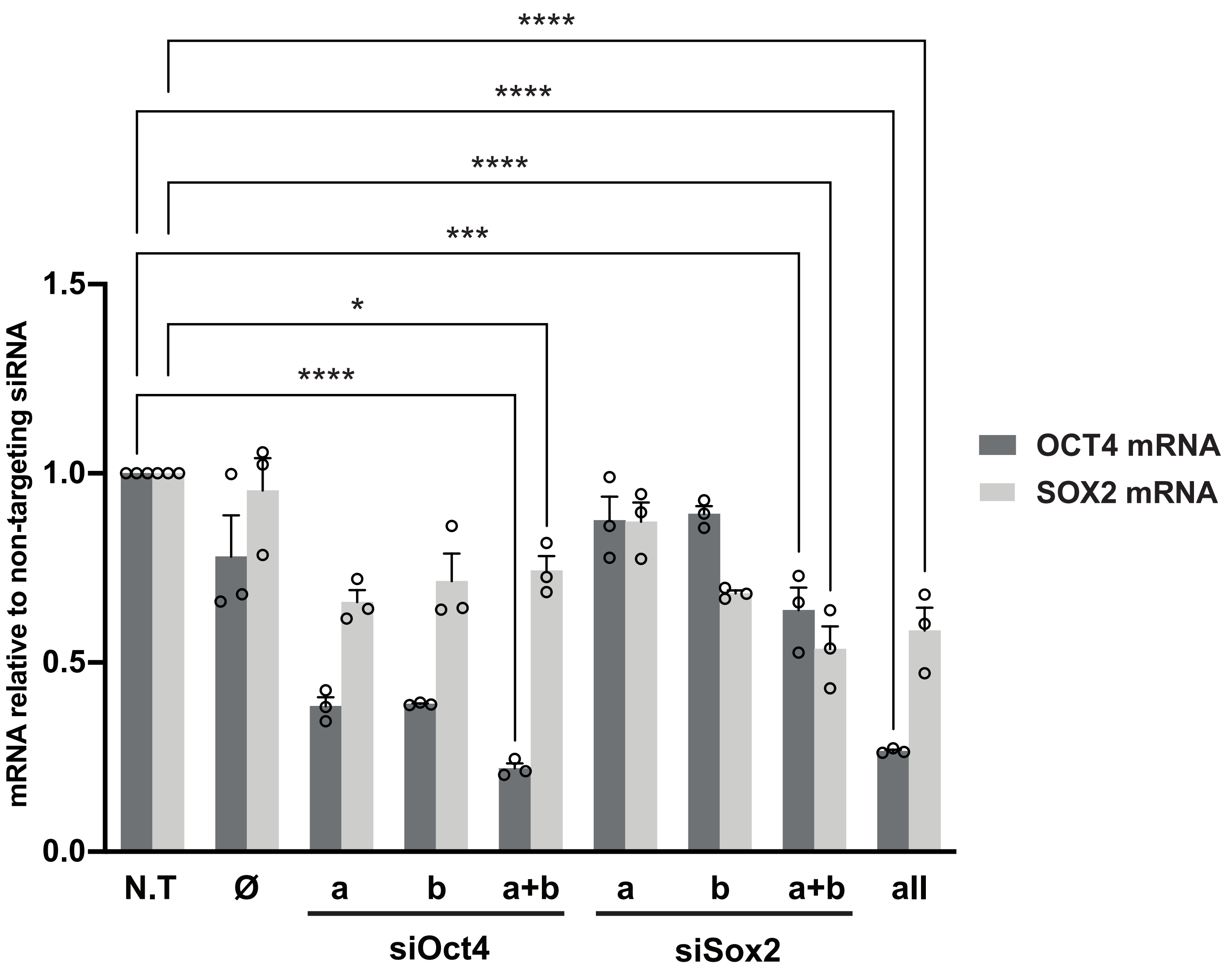

b

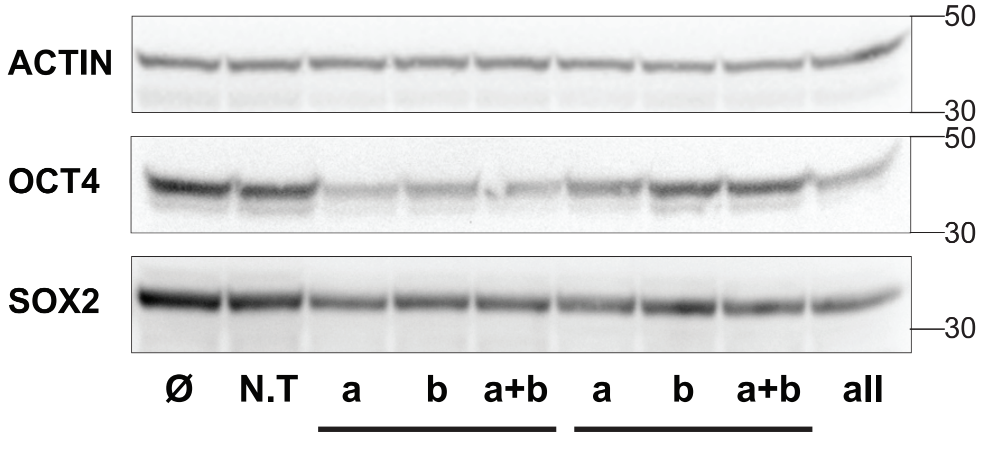

Supplementary Figure 6

Supplementary Figure 6. siRNA knockdown of OCT4 and SOX2 in ESCs containing FR1 with WT OCT4SOX2 motif, +SssI condition.

(a) Reduction of OCT4 and SOX2 mRNA levels normalized to expression of the reference housekeeping gene Snrpd3, 72h post siRNA transfection. Results are presented as mean + SD of n=3 biologically independent replicates. P-values (2-way ANOVA, *p<0.05, **p<0.01, ***p<0.001, ****p<0.0001): Oct4mRNA_siOct4a+b p=1.79E-09, Sox2mRNA_siOct4a+b p=0.0228, Oct4mRNA_siSox2a+b p=0.0004, Sox2mRNA_siSox2a+b p=4.71E-06, Oct4mRNA_siAll p=9.49E-11, Sox2mRNA_siAll p=3.60E-05. **(b)** Reduction of OCT4 and SOX2 protein levels, 72h post siRNA transfection. Western blot was performed on one of the three biologically independent knockdown experiments. Actin housekeeping gene is used as a loading control “All”: siOCT4a+siOCT4b+siSOX2a+siSOX2b. N.T: non targeting siRNA. Ø: non-transfected. For both panels, source data and uncropped blots are provided as a Source Data file.

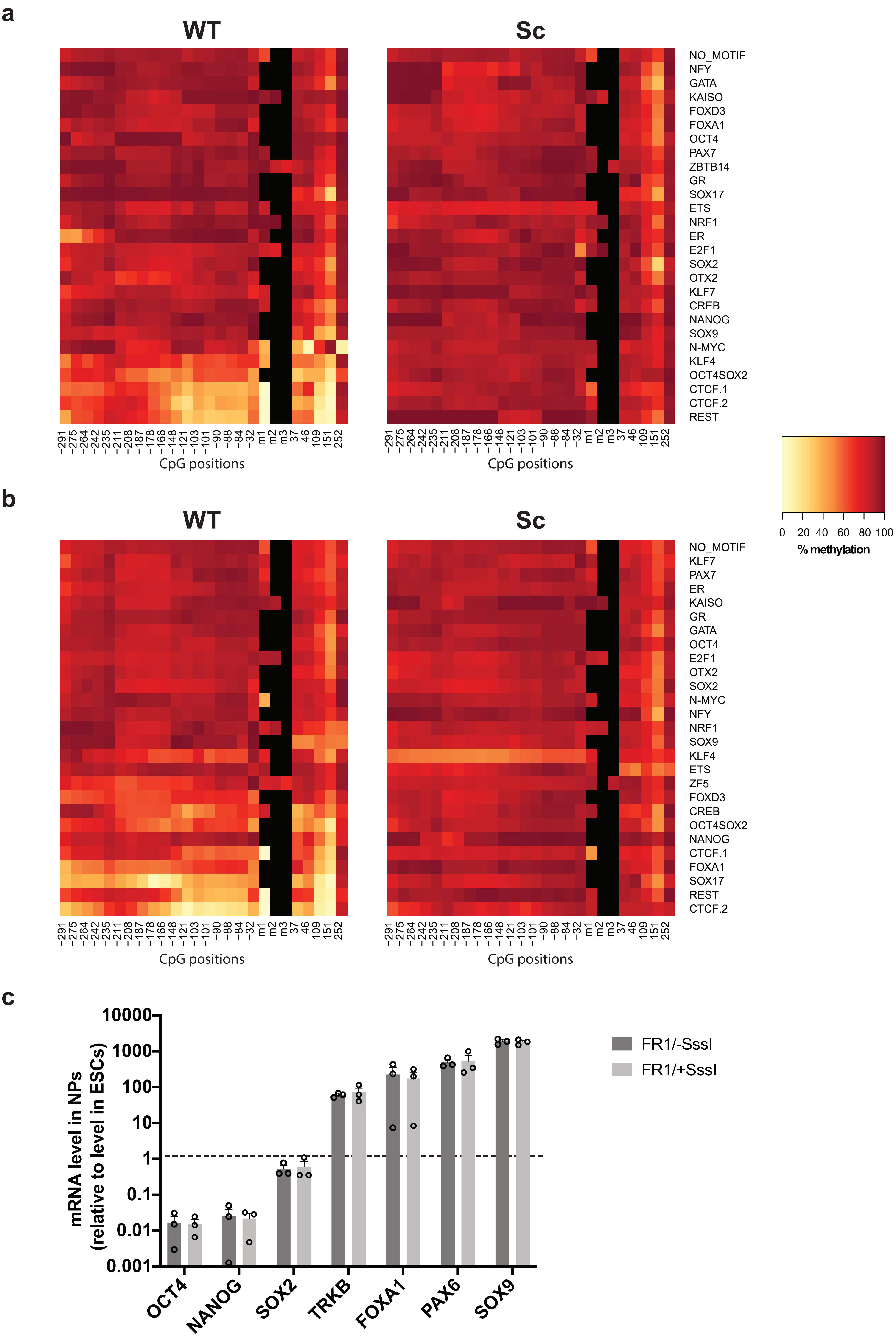

Supplementary Figure 7

Supplementary Figure 7. Differentiation into neuronal progenitors.

(**a**, **b**) Heatmaps representing methylation percentages around WT and Sc motifs in the FR1/-SssI condition (**a**) and FR1/+SssI (**b**). (**c**) qPCR validation of NP differentiation by analysis of ESC-specific (Oct4, Nanog and Sox2) and NP-specific markers (FoxA1, Pax6 and Sox9). Data are shown as mean+SEM or n=3 biologically independent experiments. Source data are provided as a Source data file.

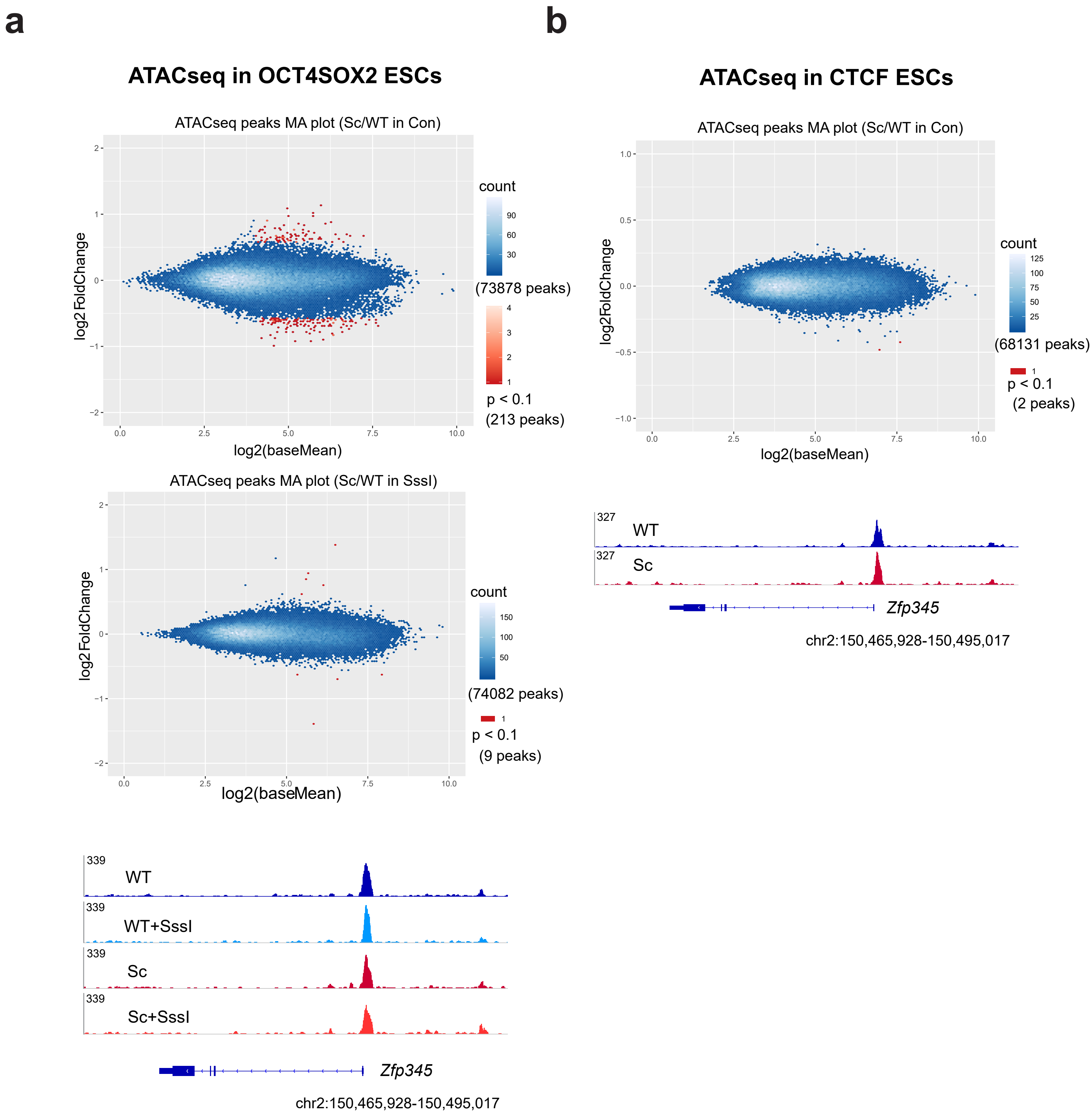

Supplementary Figure 8

Supplementary Figure 8. ATAC-Seq in ESCs at OCT4SOX2 and CTCF binding sites in FR1.

(**a**,**b**) MA plots following analysis of differential ATAC-seq peaks using the DESeq2 analysis software. As expected, there are very few changes in chromatin accessibility across the genome of the (**a**) FR1 OCT4SOX2 ESCs (WT and Sc, +/- SssI) and (**b**) FR1 CTCF ESCs (WT and Sc, -SssI). Genome browser tracks (mean values across replicates) of the ATAC-seq data for all conditions at the *Zfp345* locus.

a

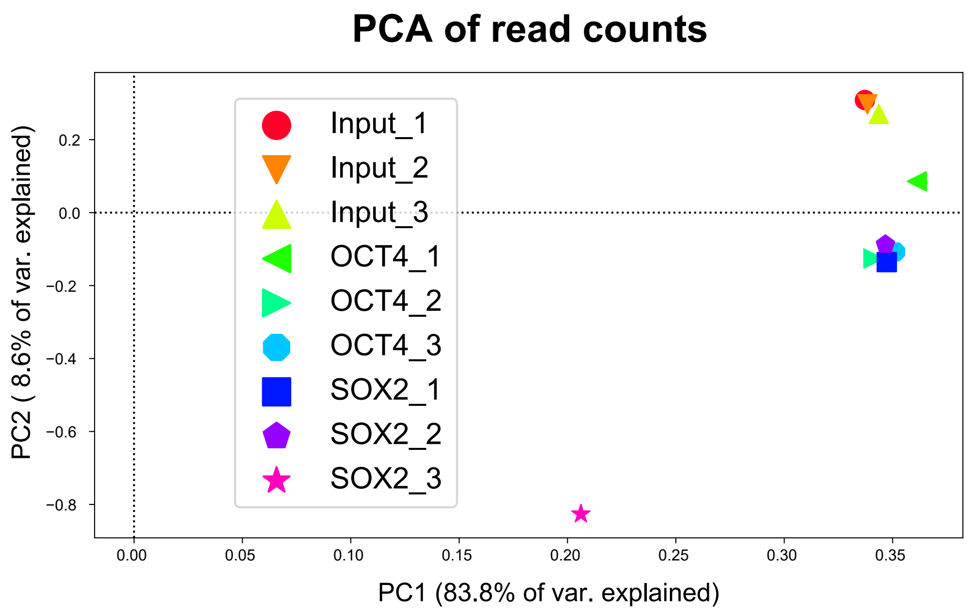

b

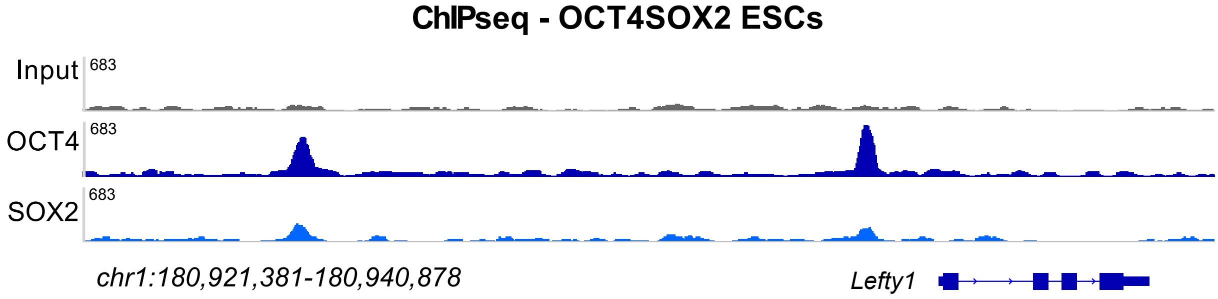

Supplementary Figure 9

Supplementary Figure 9. ChIP-Seq for OCT4 and SOX2 in ESCs.

(**a**) PCA analysis of the Oct4 and Sox2 ChIPseq data from FR1 OCT4SOX2 ESCs. As shown, the third replicate of the Sox2 ChIP is of poor quality and was therefore not used for the subsequent analysis of TF binding at the FR1 locus. (**b**) Representative genome browser tracks (mean values across replicates) of the ChIPseq data across the *Lefty1* locus.

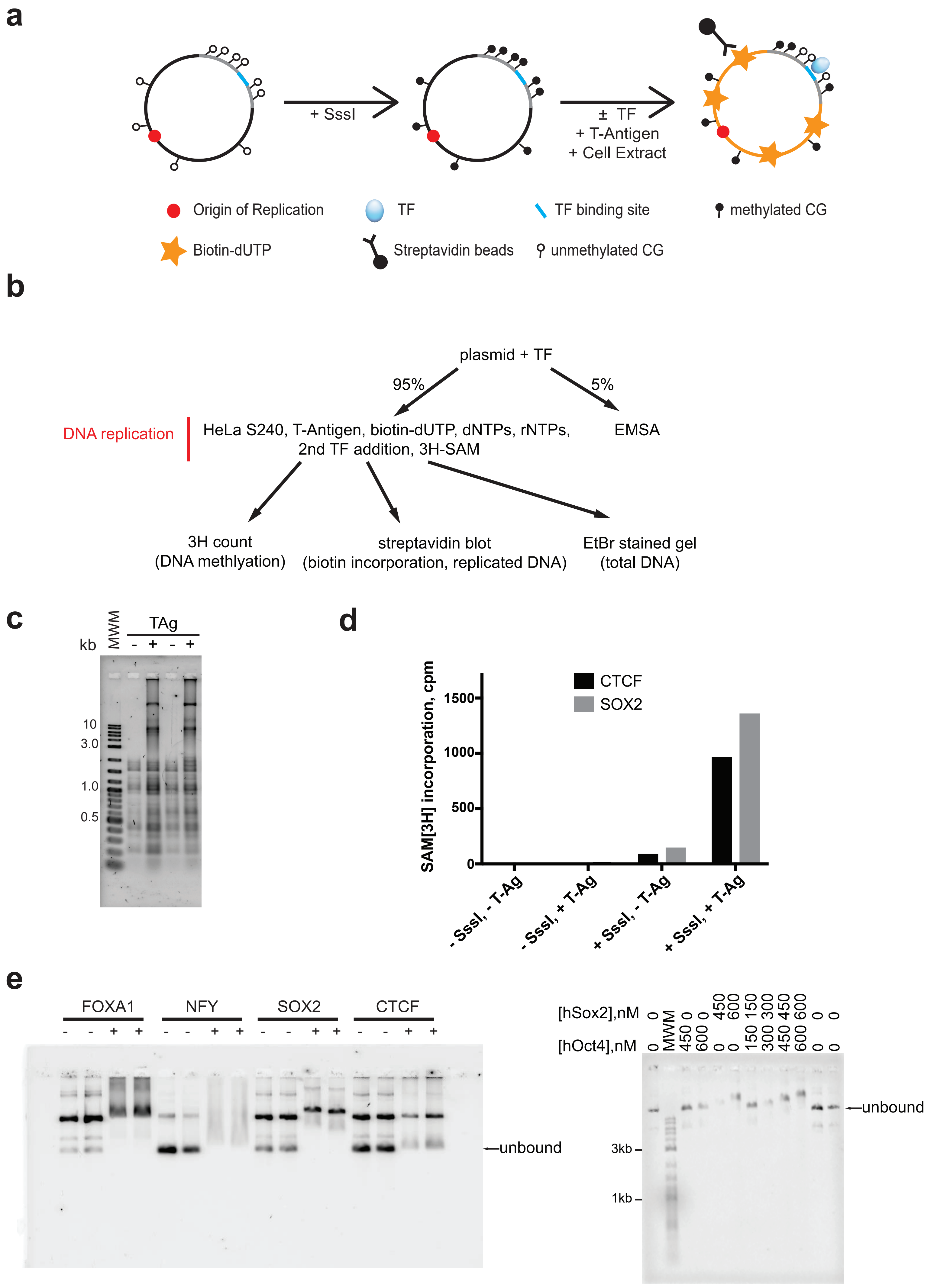

Supplementary Figure 10

Supplementary Figure 10. *In vitro* replication assay.

(**a-b**) Schematic representation of the *in vitro* replication assay. FR1 or FR2 bacterial DNA fragments are cloned into the SV40 replication vector containing an origin of replication. The resulting plasmid is *in vitro* methylated using the SssI methyltransferase. Plasmid is then incubated with the TF of interest, then replication occurs in the presence of HeLa S240 cell extract, T-Antigen, biotin-dUTP, dNTPs, rNTPs, TF and 3H-SAM. Biotinylated, replicated DNA is purified by immunoprecipitation using streptavidin beads. (**c**) DpnI digestion of the non-replicated plasmid is used to assess the completion of the replication reaction in all DNA replication experiments. (**d**) DNA methylation is maintained during *in vitro* DNA replication. –SssI and +SssI plasmids were incubated in HeLa extracts in the presence of SAM[3H] and in the presence or absence of T-Ag. DNA was purified and SAM[3H] measured by scintillation counting. Graph shows CPM - background. (**e**) 5% of the reaction is used to assess TF binding by EMSA with titrations covering a broad concentration range for all DNA replication experiments. In all cases, binding was confirmed. For all panels, source data are provided as a Source data file.

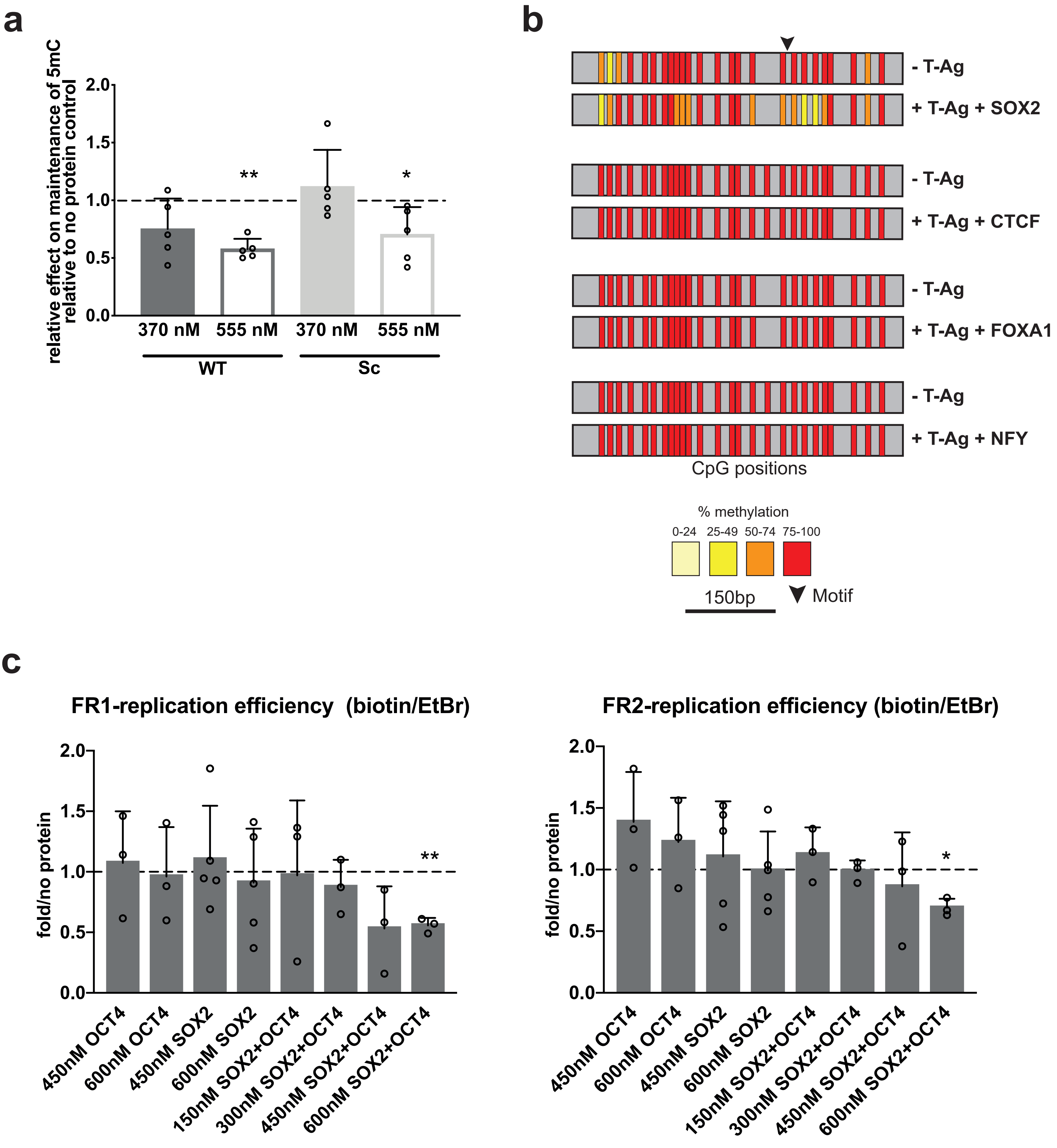

Supplementary Figure 11

Supplementary Figure 11. SOX2 inhibits DNMT1-dependent maintenance of DNA methylation during replication

(**a**) *In vitro* replication assay to assess the effect of SOX2 binding to FR1 containing SOX2-only motif on the DNMT1-dependent maintenance of DNA methylation during replication. Methylation levels following replication are measured based on the integration of radioactively labeled methyl group during replication. Results are presented as mean + SD of n=5biological replicates and analyzed as radioactive signal in the presence of SOX2 relative to the signal in absence of SOX2. P values (one sample t-test, *p<0.05, **p<0.001): SOX2_WT 370nM p=0.1026, 555nM p=0.0004; SOX2_Sc 370nM p=0.4521, 555nM=0.0491, two-tailed unpaired t-test. (**b**) Bisulfite Sanger sequencing analysis of the FR1 containing SOX2, CTCF, FOXA1 or NFY motif in absence or presence of replication (+/- T-Ag) and the PF (+/- PF, 555nM). Vertical bars correspond to CpG positions, and the color code corresponds to the percentage of methylation calculated for each CpG with a minimum coverage of 10 bisulfite reads. (**c**) Replication efficiency of FR1 and FR2 probes containing OCT4SOX2 motif as measured by the ratio of replicated DNA (biotin) to total DNA extracted from the reaction (EtBr). Results are presented as mean + SD of n=5 (hSOX2 samples) or n=3biologically independent replicates (hOCT4 and hOCT4+hSOX2 samples). The small decrease in DNA replication with high concentrations of TFs was not statistically significant. Because we calculate the ratio of incorporated SAM[3H] (DNA methylation) to incorporated biotin-dUTP (DNA replication), these differences will not affect the interpretation (**figure 7**) P-values (two-tailed unpaired t-test, **p<0.01, *p<0.05): FR1_OCT4+SOX2 600nM: 0.0063, FR2_OCT4+SOX2 600nM: 0.0179. For all panels, source data are provided as a Source data file.

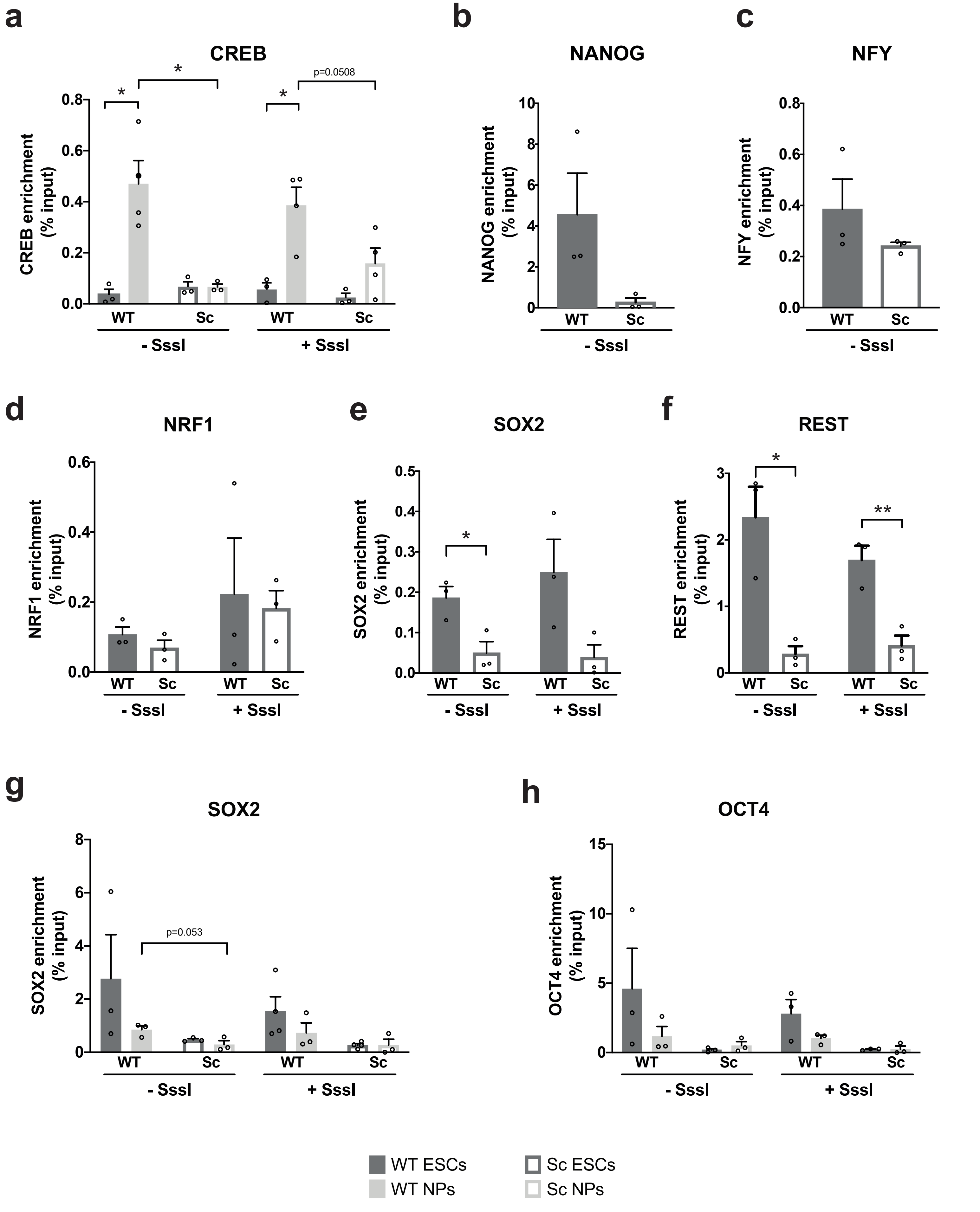

Supplementary Figure 12

Supplementary Figure 12. ChIP validation of PF binding

(**a-f**) Chromatin immunoprecipitation (ChIP) to validate CREB (**a**), NANOG (**b**), NFY (**c**), NRF1 (**d**), SOX2 (**e**) and REST (**f**) binding in ESCs and NPs. ESCs containing FR1 and individual motifs were isolated from the pool of transfected cells. When indicated, ESCs were differentiated into NPs. Results are shown as mean + SEM of n=4 (CREB_NP_WT/±SssI, CREB_NP_Sc/-SssI) or n=3 (all other conditions) biologically independent replicates. Binding of selected TFs at their motifs in the FR1 fragment is indicated as % input, that is, the enrichment of IP signal (normalized over input signal) at a selected locus. Enrichment at WT sites is estimated relative to their corresponding Sc site. In most cases, enrichment at WT motif is higher than at Sc ones. Moreover, enrichment at CREB WT motif is higher in NPs than is ESCs. P-values (two-tailed unpaired t-test): CREB_WT_NPs/-SssI vs Sc_NPs/-SssI p=0.013; CREB_WT_ESCs/-SssI vs WT_NPs/-SssI p=0.01; CREB_WT_ESCs/+SssI vs WT_NPs/+SssI p=0.013; SOX2_WT/-SssI vs SOX2_Sc/-SssI p=0.026; REST_WT_ESCs/-SssI vs Sc_ESCs /-SssI p=0.012; REST_WT_ESCs/+SssI vs Sc_ESCs/+SssI p=0.008. (**g-h**) SOX2 (**g**) and OCT4 (**h**) ChIP in ESCs and NPs containing the OCT4SOX2 motif. Results are shown as mean + SEM of n=4 (SOX2_ESCs_OCT4SOX2_WT/+SssI and SOX2_ESCs_OCT4SOX2_Sc/+SssI) or n=3 (all other conditions) biologically independent replicates. Binding of selected TFs at their motifs in the FR1 fragment is indicated as % input, that is, the enrichment of IP signal (normalized over input signal) at a selected locus. Enrichment at WT sites is estimated relative to their corresponding Sc site, and enrichment in ESCs is estimated relative to enrichment in NPs. For all panels, source data are provided as a Source data file.

Supplementary Tables

Supplementary Table 1. List of PFs selected for RMCE screening. DNA binding domains are reported as indicated in the Human TFs database (http://humantfs.ccbr.utoronto.ca/)[103]. For each factor, the main references for: pioneering activity; sensitivity or binding to methylated DNA; suspected DNA demethylation capacity and/or suspected or proven interactions with TETs and DNMTs are indicated.

PF	DNA Binding Domain	Pioneering Activity	DNA methylation sensitivity	Suspected DNA demethylation capacity
CTCF	C2H2 Zinc Finger	[104]	Binds 5mC[14,51,105]	Yes[51,105]<br>Interacts with TET[47]<br>Interacts with DNMT1[46]
KAISO	C2H2 Zinc Finger	[104]	Binds 5mC[11,33,106]	
KLF4	C2H2 Zinc Finger	[12,28,40,44,73]	Binds 5mC[10,11,33]	Yes[44]
KLF7	C2H2 Zinc Finger	[104]		
REST	C2H2 Zinc Finger		Binds 5mC[14]	Yes[14,18]<br>Interacts with TET3[107]
ZBTB14	C2H2 Zinc Finger	[104]	Binds C[106]	
ER	Zinc Finger (NHR type)	[108]	Binds 5mC[11]	Yes[109]
GR	Zinc Finger (NHR type)	[108]		Yes[110]
GATA	Zinc Finger (GATA type)	[4,111,112]	Binds 5mC[10,113]	Yes[113,114]
NANOG	Homeodomain	[20,115]		Yes[20]<br>No[114]<br>Interacts with TET[45]
OTX2	Homeodomain	[20,116]		Yes[20]
OCT4	Homeodomain + POU	[12,28,40,73]	Binds 5mC[11]	No[114]
PAX7	Homeodomain + Paired box	[77,117,118]	Binds 5mC[11]	Yes[77]
SOX2	HMG/SOX	[12,28,40,73,119]		No[114]
SOX9	HMG/SOX	[120]		
SOX17	HMG/SOX			Yes[20]
ETS	ETS	[104]	Binds C[11,113,121]	
FOXA1	Forkhead (helix-turn-helix)	[3,4,122]	Binds 5mC[106]	Yes[60,123]<br>Interacts with TET1[58]
FOXD3	Forkhead (helix-turn-helix)	[61,124]		Yes[61]
CREB	Other (bZip)	[104]	Binds C[11,33,66,67]	
E2F1	Other (E2F_TDP)	[104]	Binds C[11,106,125,126]<br>Binds 5mC[10]	Interacts with DNMT1[127]

NFY	Other (CBF/NF-Y)	[104]	Binds C[11]	
NMYC	Other (bHLH)		Binds C[18,128]	No[113]
NRF1	Other (unknown)	[104]	Binds C[18,33]<br>Binds 5mC[10,33]	No[18]

Supplementary Table 2. List of WT and Sc PF motifs and barcodes.

PF	Database ID	Experimental binding evidence	Main publications	WT motif	WT barcode	Sc motif	Sc barcode
CREB	MA0018_CREB1	HT-SELEX; compiled	11,67	TGACGTCA	tagaga	AATCGGTC	aatgta
CTCF_1	MA0139.1_CTCF_rev	ChIP-Seq	14,129	ATAGCGCCCCCTAGTGGCCA	tgagga	CCCACGGGTGGCCAACCATT	tgatga
CTCF_2	MA0139.1_CTCF			TGGCCACCAGGGGGCGCTA	tgagga	GGCAGGGAGGACCCCGTTC	tgatga
E2F1	MA0024.1_E2F1 + Chen 2008	ChIP-Seq	24	TTTCGCGC	atgtga	TCGCTTCG	aaggag
ER	MA0112_ESR1	ChIP-Seq, HT-SELEX	11,130	AAGGTCACGGTGACCTG	agaagt	GTAACTCCGAGTGAGGC	aataga
ETS	MA0098.3_ETS1	HT-SELEX, protein binding microarrays	131,132	ACCGGAAGTG	atgggg	GCCGAAAGTG	atggtt
FOXA1	MA0148.3_FOXA1	ChIP-Seq, HT-SELEX	11,133	TCCATGTTTACTTTG	aaagtg	TATTGGTCTCTATTC	ttgtga
FOXD3	MA0041.1_Foxd3	ChIP-Seq (partially), SELEX	131,134	GAATGTTTGTTT	tgagaa	ATTAGTTTGTGT	aagtgt
GATA	MA0482.1_Gata4	ChIP-Seq, HT-SELEX	11,135	TCTTATCTCCC	agtagg	CTTCACTTCCT	tgggat
GR	MA0113.3_NR3C1	SELEX	131	GGGTACATAATGTTCCC	atgtta	CACCTAGAAGTGTGCTT	atgtgg
KAISO	MA0527.1_ZBTB33	ChIP-Seq	136	CTCTCGCGAGATCTG	aatatg	GTTTCGCGGACTCAC	agaggg
KLF4	MA0039.2_Klf4	ChIP-Seq	24	TGGGCGGGGC	tgggag	GGTCCGGGGG	tatgtg
KLF7	UniProbe ID UP00093	HT-SELEX	11	ACGCCC	tagtgt	CCGCAC	tttgaa
NANOG	TRANSFAC M01123	ChIP-Seq		GGGCCCATTTCC	ttaagg	CCTAGGTCCCTG	tataga
NFY	MA0060.2_NFYA	ChIP-Seq	60	AGAGTGCTGATTGGTCCA	aaaaga	TTGGGAGTACTGGTACAC	tgtggg
NMYC	MA0104.3_Mycn	ChIP-Seq	51	GCCACGTG	aaaaaa	CATGCGGC	tagtat
NRF1	MA0506.1_NRF1	ChIP-Seq	17	GCGCCTGCGCA	agatat	TCGAGCCCGGC	tgtatt
OCT4-SOX2	MA0142.1_Pou5f1:Sox2	ChIP-Seq	51	CTTTGTTATGCAAAT	aagagt	ATACTGTGAATCTTT	tgaaag
OTX2	UniProbe ID UP00267	PBM, HT-SELEX, ChIP-Seq	8,61,62	TTAATCCC	ttttaa	ATATCCCT	tgaaga

POU5F1 (OCT4)	MA1115.1_POU5F1	ChIP-Seq	15	ATTTGCAT	aatggt	TTTAACTG	tgtgag
PAX7	MA0680.1_PAX7	HT-SELEX	53	TAATCGATTA	atagga	TATACGATAT	tggata
REST	MA0138.2_REST	ChIP-Seq	55,63	TTCAGCACCATGG ACAGCGCC	aaaagt	CAGTTACAGCCCC CAGTCGGA	tgggaa
SOX17	MA0078.1_Sox17	EMSA	64	TTCATTGTC	tattat	TTTCAGTCT	ttgggt
SOX2	MA0143.3_Sox2	ChIP-Seq	15	CCTTTGTT	ttagtg	TCTGTCTT	tagaag
SOX9	Liu 2015	ChIP-Seq	65	ACAAAGGGCCCTT TGT	ttgtta	GGCTAGTCTCATG CAA	tagata
ZBTB14	UniProbe ID UP00065	PBM	66	GCGCGCG	atttaa	CCGGGCG	tttggt

Supplementary Table 3. Table summarizing identified PPFs and SPFs in ESCs and NPs.

	**ESCs**	**NPs**
**PPFs**	CTCF<br>REST<br>KLF4<br>KLF7<br>SOX2<br>SOX9<br>NRF1<br>OTX2<br>E2F1	CTCF<br>REST<br>KLF4<br>SOX2<br>SOX9<br>N-MYC
**SPFs**	CTCF<br>REST<br>KLF4<br>SOX2<br>SOX9<br>SOX17<br>E2F1<br>N-MYC<br>GR	CTCF<br>REST<br>SOX2<br>SOX17<br>CREB<br>FOXA1<br>FOXD3

Supplementary Table 4. List of primers used in this study.

Target	Purpose	Forward	Reverse
FR1 upstream	RMCE exchange	CCTCTGGGTAAATTTGGAACA	GCAGAACGCCTGAAAAACTC
FR1 downstream	RMCE exchange	CACCGAAAGCAGACAAACCT	AACGCCTGAAAAACTCAGGA
RMCE cassette	RMCE cassette insertion upstream	AGCAAAGGTGTTCTCATATGTCA	CAAGTGGGCAGTTTACCGTA
RMCE cassette	RMCE cassette insertion downstream	TGCACGTCTTTATCCTGGATT	GGTTTAGTCTTCTCTGTGCCT
FR1_ChIP	qPCR	ACCATGAAAGTATCAGTTCCAGGC	GTGTAAGCTCTCAACCTTAAGCA
Snrpd3	qPCR	TCTCGCCTTCGCCTTCTAAC	GGACTCTTCCCGGGCAATTA
OCT4	qPCR	ATGCCGTGAAGTTGGAGAAG	GCTTGGCAAACTGTTCTAGCT
Nanog	qPCR	TTGCTTACAAGGGTCTGCTACT	ACTGGTAGAAGAATCAGGGCT
Foxa1	qPCR	GCATGAGAGCAACGACTGG	CAGGCCGGAGTTCATGTTG
Sox9	qPCR	CAGACCAGTACCCGCATCTG	AAGGGTCTCTTCTCGCTCTC
Trkb	qPCR	GGCATTCCCGAGGTTGGA	CTGGTTTGCAATGAGAATTTCCG
Pax6	qPCR	CACCAGACTCACCTGACACC	TCACTCCGCTGTGACTGTTC
Sox2	qPCR	TAGAGCTAGACTCCGGGCGATGA	TTGCCTTAAACAAGACCACGAAA
FR1 upstream	Bisulfite PCR	AAAATTTAGGAGGTAGATAATGAGGATA	CCCCTTTAATAACAACCCAATTC
FR1 downstream	Bisulfite PCR	ATTTGAAGGGAAAGGATTAGTATGT	ACCATTAAAAAAATTTTTAAACTCTTATAC
Mm_Snrpd3	qRT-PCR	TCTCGCCTTCGCCTTCTAAC	GGACTCTTCCCGGGCAATTA
Mm_SOX2 (Chen et al, PNAS, 2006)	qRT-PCR	GGCAGCTACAGCATGATGCAGGAGC	CTGGTCATGGAGTTGTACTGCAGG
Mm_OCT4	qRT-PCR	ATGCCGTGAAGTTGGAGAAG	GCTTGGCAAACTGTTCTAGCT
Intergenic	ATAC-qPCR	GGACAGACATCTGCCAAGGT	ATGCCCCTCAGCTATCACAC
FR1	ATAC-qPCR	CACCGAAAGCAGACAAACCTG	TGTATGAGCGCACAATAGCCA

Supplementary Table 5. Hi-TransMet library preparation primers. UMI-primers in step 1, where is N is random nucleotide and X indicates the barcode sequence.

**Step**	**Target**	**Sequence**
1	FR1	AATGTACAGTATTGCGTTTTXXXXXXNNNNNNNNCCCCTTTAATAACAACCCAATTC
2	Upstream Forward	TTCTTAGCGTATTGGAGTCCAAAATTTAGGAGGTAGATAATGAGGATA
	Downstream Forward	TTCTTAGCGTATTGGAGTCCATTTGAAGGGAAAGGATTAGTATGT
	Reverse	AATGTACAGTATTGCGTTTTG
3	Forward with adapter	CAAGCAGAAGACGGCATACGAGATACATCGGTGACTGGAGTTCAGACGTGTGCTCTTCCGATCTNNNTTCTTAGCGTATTGGAGTCC
	Reverse with Adapter	AATGATACGGCGACCACCGAGATCTACACTCTTTCCCTACACGACGCTCTTCCGATCTGAATGTACAGTATTGCGTTTTG
		AATGATACGGCGACCACCGAGATCTACACTCTTTCCCTACACGACGCTCTTCCGATCTTGAATGTACAGTATTGCGTTTTG
		AATGATACGGCGACCACCGAGATCTACACTCTTTCCCTACACGACGCTCTTCCGATCTCTGAATGTACAGTATTGCGTTTTG
		AATGATACGGCGACCACCGAGATCTACACTCTTTCCCTACACGACGCTCTTCCGATCTAATGTACAGTATTGCGTTTTG

Supplementary references

1. Lambert, S. A. *et al.* The Human Transcription Factors. *Cell* **172**, 650–665 (2018).
2. Sherwood, R. I. *et al.* Discovery of directional and nondirectional pioneer transcription factors by modeling DNase profile magnitude and shape. *Nat. Biotechnol.* **32**, 171–178 (2014).
3. Stadler, M. B. *et al.* DNA-binding factors shape the mouse methylome at distal regulatory regions. *Nature* **480**, 490–495 (2011).
4. Feldmann, A. *et al.* Transcription Factor Occupancy Can Mediate Active Turnover of DNA Methylation at Regulatory Regions. *PLoS Genet.* **9**, e1003994 (2013).
5. Rodriguez, C. *et al.* CTCF is a DNA methylation-sensitive positive regulator of the INK/ARF locus. *Biochem. Biophys. Res. Commun.* **392**, 129–134 (2010).
6. Dubois-Chevalier, J. *et al.* A dynamic CTCF chromatin binding landscape promotes DNA hydroxymethylation and transcriptional induction of adipocyte differentiation. *Nucleic Acids Res.* **42**, 10943–10959 (2014).
7. Zampieri, M. *et al.* ADP-ribose polymers localized on Ctcf–Parp1–Dnmt1 complex prevent methylation of Ctcf target sites. *Biochem. J.* **441**, 645–652 (2012).
8. Yin, Y. *et al.* Impact of cytosine methylation on DNA binding specificities of human transcription factors. *Science* **356**, eaaj2239 (2017).
9. Spruijt, C. G. *et al.* Dynamic Readers for 5-(Hydroxy)Methylcytosine and Its Oxidized Derivatives. *Cell* **152**, 1146–1159 (2013).
10. Bartke, T. *et al.* Nucleosome-Interacting Proteins Regulated by DNA and Histone Methylation. *Cell* **143**, 470–484 (2010).
11. Takahashi, K. & Yamanaka, S. Induction of Pluripotent Stem Cells from Mouse Embryonic and Adult Fibroblast Cultures by Defined Factors. *Cell* **126**, 663–676 (2006).

12. Soufi, A. *et al.* Pioneer Transcription Factors Target Partial DNA Motifs on Nucleosomes to Initiate Reprogramming. *Cell* **161**, 555–568 (2015).
13. Chronis, C. *et al.* Cooperative Binding of Transcription Factors Orchestrates Reprogramming. *Cell* **168**, 442-459.e20 (2017).
14. Sardina, J. L. *et al.* Transcription Factors Drive Tet2-Mediated Enhancer Demethylation to Reprogram Cell Fate. 17 (2018).
15. Soufi, A., Donahue, G. & Zaret, K. S. Facilitators and Impediments of the Pluripotency Reprogramming Factors' Initial Engagement with the Genome. *Cell* **151**, 994–1004 (2012).
16. Hu, S. *et al.* DNA methylation presents distinct binding sites for human transcription factors. *eLife* **2**, e00726 (2013).
17. Domcke, S. *et al.* Competition between DNA methylation and transcription factors determines binding of NRF1. *Nature* **528**, 575–579 (2015).
18. Perera, A. *et al.* TET3 Is Recruited by REST for Context-Specific Hydroxymethylation and Induction of Gene Expression. *Cell Rep.* **11**, 283–294 (2015).
19. Swinstead, E. E. *et al.* Steroid Receptors Reprogram FoxA1 Occupancy through Dynamic Chromatin Transitions. *Cell* **165**, 593–605 (2016).
20. Dumasia, K., Kumar, A., Deshpande, S. & Balasinor, N. H. Estrogen signaling, through estrogen receptor β, regulates DNA methylation and its machinery in male germ line in adult rats. *Epigenetics* **12**, 476–483 (2017).
21. Thomassin, H. Glucocorticoid-induced DNA demethylation and gene memory during development. *EMBO J.* **20**, 1974–1983 (2001).
22. Cirillo, L. A. *et al.* Opening of Compacted Chromatin by Early Developmental Transcription Factors HNF3 (FoxA) and GATA-4. *Mol. Cell* **9**, 279–289 (2002).
23. Huang, P. *et al.* Induction of functional hepatocyte-like cells from mouse fibroblasts

by defined factors. *Nature* **475**, 386–389 (2011).

24. Sekiya, S. & Suzuki, A. Direct conversion of mouse fibroblasts to hepatocyte-like cells by defined factors. *Nature* **475**, 390–393 (2011).
25. Lea, A. J. *et al.* Genome-wide quantification of the effects of DNA methylation on human gene regulation. *eLife* **7**, e37513 (2018).
26. Suzuki, T. *et al.* A screening system to identify transcription factors that induce binding site-directed DNA demethylation. *Epigenetics Chromatin* **10**, 60 (2017).
27. Tsankov, A. M. *et al.* Transcription factor binding dynamics during human ES cell differentiation. *Nature* **518**, 344–349 (2015).
28. Lee, M. T. *et al.* Nanog, Pou5f1 and SoxB1 activate zygotic gene expression during the maternal-to-zygotic transition. *Nature* **503**, 360–364 (2013).
29. Costa, Y. *et al.* NANOG-dependent function of TET1 and TET2 in establishment of pluripotency. *Nature* **495**, 370–374 (2013).
30. Boulay, G. *et al.* OTX2 Activity at Distal Regulatory Elements Shapes the Chromatin Landscape of Group 3 Medulloblastoma. 15.
31. Budry, L. *et al.* The selector gene Pax7 dictates alternate pituitary cell fates through its pioneer action on chromatin remodeling. *Genes Dev.* **26**, 2299–2310 (2012).
32. Mayran, A. *et al.* Pioneer factor Pax7 deploys a stable enhancer repertoire for specification of cell fate. *Nat. Genet.* **50**, 259–269 (2018).
33. McKinnell, I. W. *et al.* Pax7 activates myogenic genes by recruitment of a histone methyltransferase complex. *Nat. Cell Biol.* **10**, 77–84 (2008).
34. Velychko, S. *et al.* Excluding Oct4 from Yamanaka Cocktail Unleashes the Developmental Potential of iPSCs. *Cell Stem Cell* **25**, 737-753.e4 (2019).
35. Adam, R. C. *et al.* Pioneer factors govern super-enhancer dynamics in stem cell plasticity and lineage choice. *Nature* **521**, 366–370 (2015).

36. Gaston, K. & Fried, M. CpG methylation has differential effects on the binding of YY1 and ETS proteins to the bi-directional promoter of the Surf-1 and Surf-2 genes. 9.
37. Cirillo, L. A. & Zaret, K. S. An Early Developmental Transcription Factor Complex that Is More Stable on Nucleosome Core Particles Than on Free DNA. *Mol. Cell* **4**, 961–969 (1999).
38. Cirillo, L. A. Binding of the winged-helix transcription factor HNF3 to a linker histone site on the nucleosome. *EMBO J.* **17**, 244–254 (1998).
39. Zhang, Y. Nucleation of DNA repair factors by FOXA1 links DNA demethylation to transcriptional pioneering. *Nat. Genet.* **48**, 13 (2016).
40. Donaghey, J. *et al.* Genetic determinants and epigenetic effects of pioneer-factor occupancy. *Nat. Genet.* **50**, 250–258 (2018).
41. Yang, Y. A. *et al.* FOXA1 potentiates lineage-specific enhancer activation through modulating TET1 expression and function. *Nucleic Acids Res.* **44**, 8153–8164 (2016).
42. Xu, J. *et al.* Transcriptional competence and the active marking of tissue-specific enhancers by defined transcription factors in embryonic and induced pluripotent stem cells. *Genes Dev.* **23**, 2824–2838 (2009).
43. Lukoseviciute, M. *et al.* From Pioneer to Repressor: Bimodal foxd3 Activity Dynamically Remodels Neural Crest Regulatory Landscape In Vivo. *Dev. Cell* **47**, 608-628.e6 (2018).
44. Zhang, X. *et al.* Genome-wide analysis of cAMP-response element binding protein occupancy, phosphorylation, and target gene activation in human tissues. *Proc. Natl. Acad. Sci.* **102**, 4459–4464 (2005).
45. Mann, I. K. *et al.* CG methylated microarrays identify a novel methylated sequence bound by the CEBPB ATF4 heterodimer that is active in vivo. *Genome Res.* **23**, 988–997 (2013).

46. Kovesdi, I., Reichel, R. & Nevins, J. R. Role of an adenovirus E2 promoter binding factor in ElA-mediated coordinate gene control. *Proc Natl Acad Sci USA* 5 (1987).

47. Campanero, M. R., Armstrong, M. I. & Flemington, E. K. CpG methylation as a mechanism for the regulation of E2F activity. *Proc. Natl. Acad. Sci.* **97**, 6481–6486 (2000).

48. Robertson, K. D. *et al.* DNMT1 forms a complex with Rb, E2F1 and HDAC1 and represses transcription from E2F-responsive promoters. *Nat. Genet.* **25**, 338–342 (2000).

49. Perini, G., Diolaiti, D., Porro, A. & Della Valle, G. In vivo transcriptional regulation of N-Myc target genes is controlled by E-box methylation. *Proc. Natl. Acad. Sci.* **102**, 12117–12122 (2005).

50. Barski, A. *et al.* High-Resolution Profiling of Histone Methylations in the Human Genome. *Cell* **129**, 823–837 (2007).

51. Chen, X. *et al.* Integration of External Signaling Pathways with the Core Transcriptional Network in Embryonic Stem Cells. *Cell* **133**, 1106–1117 (2008).

52. Welboren, W.-J. *et al.* ChIP-Seq of ERα and RNA polymerase II defines genes differentially responding to ligands. *EMBO J.* **28**, 1418–1428 (2009).

53. Jolma, A. *et al.* DNA-Binding Specificities of Human Transcription Factors. *Cell* **152**, 327–339 (2013).

54. Wei, G.-H. *et al.* Genome-wide analysis of ETS-family DNA-binding in vitro and in vivo. *EMBO J.* **29**, 2147–2160 (2010).

55. The ENCODE Project Consortium. An integrated encyclopedia of DNA elements in the human genome. *Nature* **489**, 57–74 (2012).

56. Respuela, P. *et al.* Foxd3 Promotes Exit from Naive Pluripotency through Enhancer Decommissioning and Inhibits Germline Specification. *Cell Stem Cell* **18**, 118–133

(2016).

57. Ang, Y.-S. *et al.* Disease Model of GATA4 Mutation Reveals Transcription Factor Cooperativity in Human Cardiogenesis. *Cell* **167**, 1734-1749.e22 (2016).
58. Blattler, A. *et al.* ZBTB33 binds unmethylated regions of the genome associated with actively expressed genes. *Epigenetics Chromatin* **6**, 13 (2013).
59. Balwierz, P. J. *et al.* ISMARA: automated modeling of genomic signals as a democracy of regulatory motifs. *Genome Res.* **24**, 869–884 (2014).
60. Oldfield, A. J. *et al.* Histone-Fold Domain Protein NF-Y Promotes Chromatin Accessibility for Cell Type-Specific Master Transcription Factors. *Mol. Cell* **55**, 708–722 (2014).
61. Berger, M. F. *et al.* Variation in Homeodomain DNA Binding Revealed by High-Resolution Analysis of Sequence Preferences. *Cell* **133**, 1266–1276 (2008).
62. Samuel, A., Housset, M., Fant, B. & Lamonerie, T. Otx2 ChIP-seq Reveals Unique and Redundant Functions in the Mature Mouse Retina. *PLoS ONE* **9**, e89110 (2014).
63. Johnson, D. S., Mortazavi, A., Myers, R. M. & Wold, B. Genome-Wide Mapping of in Vivo Protein-DNA Interactions. *Science* **316**, 1497–1502 (2007).
64. Kanai, Y. *et al.* Identification of Two Soxl7 Messenger RNA Isoforms, with and without the High Mobility Group Box Region, and Their Differential Expression in Mouse Spermatogenesis. *J. Cell Biol.* **133**, 15 (1996).
65. Liu, C.-F. & Lefebvre, V. The transcription factors SOX9 and SOX5/SOX6 cooperate genome-wide through super-enhancers to drive chondrogenesis. *Nucleic Acids Res.* **43**, 8183–8203 (2015).
66. Badis, G. *et al.* Diversity and Complexity in DNA Recognition by Transcription Factors. **324**, 5 (2009).
