# Supplementary material for: High throughput screening identifies SOX2 as a super pioneer factor that inhibits DNA methylation maintenance at its binding sites
Source: Nat Commun. 2021 Jun 7;12:3337. doi: 10.1038/s41467-021-23630-x (PMC8184831; doi:10.1038/s41467-021-23630-x)
Supplement: Supplementary file 3 — Reporting Summary [file 41467_2021_23630_MOESM3_ESM.pdf]

nature research

Corresponding author(s): Rabih Murr

Last updated by author(s): Mar 17, 2021

Reporting Summary

Nature Research wishes to improve the reproducibility of the work that we publish. This form provides structure for consistency and transparency in reporting. For further information on Nature Research policies, see our Editorial Policies and the Editorial Policy Checklist.

Statistics

For all statistical analyses, confirm that the following items are present in the figure legend, table legend, main text, or Methods section.

n/a	Confirmed	
☐	☒	The exact sample size (*n*) for each experimental group/condition, given as a discrete number and unit of measurement
☐	☒	A statement on whether measurements were taken from distinct samples or whether the same sample was measured repeatedly
☐	☒	The statistical test(s) used AND whether they are one- or two-sided<br>*Only common tests should be described solely by name; describe more complex techniques in the Methods section.*
☐	☒	A description of all covariates tested
☐	☒	A description of any assumptions or corrections, such as tests of normality and adjustment for multiple comparisons
☐	☒	A full description of the statistical parameters including central tendency (e.g. means) or other basic estimates (e.g. regression coefficient) AND variation (e.g. standard deviation) or associated estimates of uncertainty (e.g. confidence intervals)
☐	☒	For null hypothesis testing, the test statistic (e.g. *F*, *t*, *r*) with confidence intervals, effect sizes, degrees of freedom and *P* value noted<br>*Give P values as exact values whenever suitable.*
☒	☐	For Bayesian analysis, information on the choice of priors and Markov chain Monte Carlo settings
☐	☒	For hierarchical and complex designs, identification of the appropriate level for tests and full reporting of outcomes
☐	☒	Estimates of effect sizes (e.g. Cohen's *d*, Pearson's *r*), indicating how they were calculated

Our web collection on statistics for biologists contains articles on many of the points above.

Software and code

Policy information about availability of computer code

Data collection	RNA-Seq fastq were obtained using a HiSeq 4000 (SE 50bp).<br>Amplicon Bisulfite-seq fastq were obtained using a MiSeq (PE 300bp)
Data analysis	For RNA sequencing analysis, a traditional worflow was used, starting with the trimming of each fastq libraries using Trimmomatic v0.38 (ILLUMINACLIP:TruSeq3-SE:2:30:10 LEADING:3 TRAILING:3 SLIDINGWINDOW:4:15 MINLEN:36) , followed by the mapping on ensembl reference genome GRm38 v89 using STAR v2.6.1b (--sjdbOverhang 49). The extraction was performed using featurecounts from Rsubread v1.6.2 (-a GRCm38.89.gtf -F GTF -t exon -g gene_id -s 0) and the expression was normalised (RPKM). Expression levels and differential expression levels were performed on R using edgeR v3.27.0 (FC>3 and FDR>0.1%).<br>For Hi-TransMet analysis, adapters present in the R2 of each library were first removed using flexbar v3.4.0 (-r ${R2} -p ${R1} -b ${adapt} -bt 0.2 -be LEFT -qt 0). Next, libraries were trimmed using trim_galore v 0.4.4 (qscore threshold of 20). Reads were demultiplexed with flexbar according to cell-type/treatment using barcodes provided in table 1. In parallel, the Unique Molecular Identifier (UMI) is extracted using the --umi-tags option of flexbar. The reads were then filtered and sorted by motifs using vmatchpattern function from BioString v2.54.0 package (0 missmatch allowed). Reads containing a given motif were then mapped with bismark v 0.20.0 to the reference sequence including FR1 and the given motif (–non_directional -X 1000 -N 0 -q ref/${motif_name}). For embryonic stem cells and neuronal progenitors, we added a step consisting in filtering out all reads with an average non-CG conversion rate above 2%.<br>Next, to remove PCR amplification bias, we de-duplicate reads using previously extracted UMI tag with UMI-tools v0.5.5 (method=uniq).<br>We then extracted methylation status for individual CpGs in fragments containing the wild-type and scrambled motifs.<br>Hypomethylated regions (HMRs) were identified as regions of more than 50bp containing 3 consecutive CpGs having a difference of methylation higher than 10%, using DSS v2.33.0 package.We applied a smoothing option with a window range of 50bp, similar to what was used in heatmaps in figures 2-4 and supplementary figure 4.<br>All graphs were generated using the ggplot2 suite.

For manuscripts utilizing custom algorithms or software that are central to the research but not yet described in published literature, software must be made available to editors and reviewers. We strongly encourage code deposition in a community repository (e.g. GitHub). See the Nature Research guidelines for submitting code & software for further information.

Data

Policy information about availability of data

All manuscripts must include a data availability statement. This statement should provide the following information, where applicable:

- Accession codes, unique identifiers, or web links for publicly available datasets
- A list of figures that have associated raw data
- A description of any restrictions on data availability

Provide your data availability statement here.

Field-specific reporting

Please select the one below that is the best fit for your research. If you are not sure, read the appropriate sections before making your selection.

☒ Life sciences ☐ Behavioural & social sciences ☐ Ecological, evolutionary & environmental sciences

For a reference copy of the document with all sections, see nature.com/documents/nr-reporting-summary-flat.pdf

Life sciences study design

All studies must disclose on these points even when the disclosure is negative.

Sample size	For Hi-TransMet experiments, sample size was determined by the read coverage for each motif, i.e. to reach a minimum coverage of 10 for each CpG on fragments with a given TF binding motif. This minimal coverage is obtained after UMI de-duplication to avoid amplification bias. Correlation of replicates of each condition was confirmed and subsequently all replicates were combined.<br>For Sanger Bisulfite Sequencing, sample size was increased until we reached a 10 reads coverage for all CpGs. This is the standard coverage used in this type of assays.
Data exclusions	There was no exclusion/inclusion of samples or animals in the analysis. Sequencing data were filtered according to the criteria described in the methods section.
Replication	All experiments, except for Western Blot in SupFig6b were replicated independently at least 3 times. All attempts at replication were successful.
Randomization	Allocation of samples to different treatments was random.
Blinding	Investigators were not blinded during data collection or analysis.<br>Cell genotypes and plasmid identities were known before the preparation of sequencing libraries or in vitro assays, respectively. As a result, investigators knew what samples they were handling and analyzing.

Reporting for specific materials, systems and methods

We require information from authors about some types of materials, experimental systems and methods used in many studies. Here, indicate whether each material, system or method listed is relevant to your study. If you are not sure if a list item applies to your research, read the appropriate section before selecting a response.

Materials & experimental systems

n/a	Involved in the study	
☐	☒	Antibodies
☐	☒	Eukaryotic cell lines
☒	☐	Palaeontology and archaeology
☒	☐	Animals and other organisms
☒	☐	Human research participants
☒	☐	Clinical data
☒	☐	Dual use research of concern

Methods

n/a	Involved in the study	
☐	☒	ChIP-seq
☒	☐	Flow cytometry
☒	☐	MRI-based neuroimaging

Antibodies

Antibodies used	For ChIP: CTCF (Diagenode, C15410210), OCT4 (Diagenode, C15410305 and Cell Signaling Technology, 5677), SOX2 (Santa Cruz, sc-365823X), CREB (Abcam, ab31387), NRF1 (Abcam, ab55744), REST (Millipore, 17-641), NANOG (Cell Signaling Technology, 8822), NFY-A (Santa Cruz, sc-17753X).<br>For Western blotting: OCT4 (Cell Signaling Technology, 5677), SOX2 (Santa Cruz, sc-365823X), ACTIN (Abcam, ab8227), goat anti-rabbit IgG (BioRad, 170-6515) and goat anti-mouse IgG (BioRad, 170-6516).

Validation	CTCF and OCT4 antibodies were validated by Diagenode by ChIP-qPCR and ChIP Seq. OCT4 and NANOG antibodies were validated by Cell Signaling Technology by ChIP-qPCR (OCT4 and NANOG) and ChIP-Seq (NANOG). SOX2 and NRF1 antibodies were validated by Santa-Cruz by immunofluorescence, immunostaining and western blotting, and were recommended by the company for ChIP assays. Both were used in publications for ChIP. CREB and NRF1 antibodies were validated by Abcam by western blotting and were used for ChIP in publications. REST antibody was validated by Millipore by ChIP-qPCR and western blotting. Actin antibody was validated by Western blotting and immunofluorescence. Supporting documentation for secondary antibodies is available on the company's website.<br>Further validation reports could be found on the the suppliers' websites.

Eukaryotic cell lines

Policy information about cell lines

Cell line source(s)	WT mouse ES cells used are TC-1(WT) ES cells and were obtained from the lab of Dirk Schübeler (FMI, Basel) as a part of an MTA signed with Prof Ann Dean at the NIDDK, Bethesda, USA.<br>TET-TKO mouse ES cells were generated in Dawlaty et al., Dev.Cell. 2013 and kindly provided by the lab of Rudolph Jaenisch (Whitehead Institute for Biomedical Research). An MTA was signed.<br>TriExSf9-Novagen cells were purchased from Sigma, catalogue #71023.
Authentication	RNA-Seq analysis confirmed the authenticity of the cell lines used.
Mycoplasma contamination	Cell lines tested negative for mycoplasma.
Commonly misidentified lines<br>(See ICLAC register)	Cell lines used are not listed in the database of commonly misidentified lines.

ChIP-seq

Data deposition

☒ Confirm that both raw and final processed data have been deposited in a public database such as GEO.

☒ Confirm that you have deposited or provided access to graph files (e.g. BED files) for the called peaks.

Data access links<br>*May remain private before publication.*	The sequencing data were deposited in the GEO repository (GSE144524).
Files in database submission	Raw and summary data files for ChIP-seq and ATAC-seq<br>-fastq files: Input_R3_S16_L001_R1_001.fastq.gz; Input_R2_S15_L001_R1_001.fastq.gz;<br>Input_R1_S14_L001_R1_001.fastq.gz; OCT4_R1_S11_L001_R1_001.fastq; OCT4_R2_S13_L001_R1_001.fastq.gz;<br>OCT4_R3_S18_L001_R1_001.fastq.gz; SOX2_R1_S12_L001_R1_001.fastq.gz; SOX2_R2_S17_L001_R1_001.fastq.gz;<br>CTCF_WT3_S6_L002_R1_001.fastq.gz; CTCF_WT2_S5_L002_R1_001.fastq.gz; CTCF_WT1_S4_L002_R1_001.fastq.gz;<br>CTCF_SC6_S3_L002_R1_001.fastq.gz; CTCF_SC5_S2_L002_R1_001.fastq.gz; CTCF_SC4_S1_L002_R1_001.fastq.gz;<br>Oct4Sox2_WTSssI9_S18_L004_R1_001.fastq.gz; Oct4Sox2_WTSssI8_S17_L004_R1_001.fastq.gz;<br>Oct4Sox2_WTSssI7_S16_L004_R1_001.fastq.gz; Oct4Sox2_WTCon3_S15_L004_R1_001.fastq.gz;<br>Oct4Sox2_WTCon2_S14_L004_R1_001.fastq.gz; Oct4Sox2_WTCon1_S13_L004_R1_001.fastq.gz;<br>Oct4Sox2_ScSssI12_S12_L004_R1_001.fastq.gz; Oct4Sox2_ScSssI11_S11_L004_R1_001.fastq.gz;<br>Oct4Sox2_ScSssI10_S10_L004_R1_001.fastq.gz; Oct4Sox2_ScCon6_S9_L004_R1_001.fastq.gz;<br>Oct4Sox2_ScCon5_S8_L004_R1_001.fastq.gz; Oct4Sox2_ScCon4_S7_L004_R1_001.fastq.gz;<br>CTCF_ATAC_peaks.bed; Oct4Sox2_ATAC_peaks.bed
Genome browser session<br>(e.g. UCSC)	n/a

Methodology

Replicates	All sequencing data was obtained in triplicate. Note: For the Sox2 ChIPseq replicate #3 was of poor quality and not included in the analysis.
Sequencing depth	Sequencing was performed as 100 bp single-end reads. All samples contain ≥ 10 million uniquely mapped reads.
Antibodies	OCT4 (Cell Signaling Technology, 5677) and SOX2 (Santa Cruz, sc-365823X).
Peak calling parameters	Reads were aligned to the mm10 genome assembly using Bowtie 2, requiring mapping qualities > 20. Duplicate reads were discarded. Peaks were called using MACS 2.1.1 with default settings.
Data quality	Differential called peaks were only considered if the FDR-adjusted p value was < 0.10. For example, in ATAC-seq experiments performed in CTCF ESCs 68,133 peaks were detected and only 2 peaks decreased significantly when comparing WT to Sc.
Software	Bowtie2, Picard, MACS2, Bedtools, Deeptools, DESeq2 and R.
